# Reasons for Switching Antihypertensive Medication in General Practice: A Cross-Sectional Czech Nationwide Survey

**DOI:** 10.1097/MD.0000000000000168

**Published:** 2014-12-12

**Authors:** Jan Václavík, Petra Vysočanová, Jitka Seidlerová, Petr Zajíček, Ondřej Petrák, Jaroslav Dlask, Jiří Krýza

**Affiliations:** From the Department of Internal Medicine I—Cardiology, University Hospital Olomouc and Faculty of Medicine and Dentistry, Palacký University, Olomouc, Czech Republic (JV); Department of Internal Cardiology Medicine, University Hospital Brno, Brno, Czech Republic (PV); Department of Internal Medicine II, Faculty of Medicine in Pilsen, Charles University, Czech Republic (JS); Department of Internal Medicine, Valašské Meziříčí Hospital, Valašské Meziříčí, Czech Republic (PZ); Third Department of Medicine, First Faculty of Medicine, Charles University and General University Hospital, Prague, Czech Republic (OP); Boehringer Ingelheim Czech Republic (JD); and Cegedim CZ, Czech Republic (JK).

## Abstract

To improve blood pressure (BP) control of their patients, physicians either adjust or switch antihypertensive medication. Currently, there is only limited information available on why physicians decide to switch antihypertensive medications.

A questionnaire-based survey was performed between November 2011 and March 2012 in the Czech Republic. General practitioners were asked to fill in questionnaires about their hypertensive patients whose antihypertensive medication they were planning to change. These questionnaires recorded data about patient demographic information, cardiovascular risk factors, BP values, and reasons for switching antihypertensive medication.

Two hundred eight-six general practitioners surveyed a total of 4341 hypertensive patients. The mean age of the patients was 59.8 years, 68.9% of patients were overweight or obese. Uncontrolled office systolic and diastolic BP >140/90 mm Hg was present in 89.6% and 81.5% of patients, respectively, despite the fact that 49.4% of patients used a combination of 2 or more antihypertensive drugs. The most common reasons for switching medication were insufficient BP control (73.7%), followed by aiming for a better 24-hour effect (38.4%) and increased cardiovascular risk of the patients (37.7%).

The major reason for switching antihypertensive treatment in general practice was insufficient BP control. Switching medication because of adverse drug effects is less frequent than reported a decade ago.

## INTRODUCTION

Arterial hypertension is one of the most common cardiovascular disorders, with its global prevalence currently exceeding 1 billion people.^1^ Worldwide, hypertension is not adequately controlled. The rate of hypertension control (<140/90 mm Hg) was reported to be higher in the United States (63%) and Czech Republic (51%) compared with 31% to 46% in Western European and 17% to 36% in other Central and Eastern European countries.^2,3^

To improve blood pressure (BP) control, physicians often adjust antihypertensive medication by increasing drug doses, switching BP lowering drugs, or combining different classes of antihypertensives. Currently, there is only limited information available on the reasons for which physicians decide to switch antihypertensive medications.^4–6^ As no recent data are available on reasons for antihypertensive medication switching in the Czech Republic or Europe, we decided to perform this cross-sectional nationwide survey. The aim of the survey was to evaluate reasons for switching antihypertensive medication in a large number of hypertensive patients among general practitioners (GPs) in the Czech Republic.

## METHODS

### Survey Population

This survey included patients with arterial hypertension who were routinely managed by their GPs residing in the Czech Republic. It was planned to involve approximately 390 physicians, with each GP supposed to survey 15 male or female hypertensive patients in whom the change of antihypertensive medication was planned. Survey was performed between November 3, 2011, and March 30, 2012. Ethical approval is not required for this type of survey.

### Data Acquisition

GPs filled in a standardized questionnaire to obtain information on each patient's demographic data, body mass index (BMI; kg/m^2^), office BP values, personal and family medical history, lifestyle habits and presence of cardiovascular (CV) risk factors, duration of hypertension, current antihypertensive drug treatment, and reasons for changing the antihypertensive medication.

Risk factors were defined as abdominal obesity (waist circumference >102 cm in men and >88 cm in women), current cigarette smoking, insufficient physical activity, hypercholesterolemia (serum total cholesterol >5 mmol/l), albuminuria, type 2 diabetes mellitus, personal history of stroke, myocardial infarction, peripheral artery disease or heart failure, positive family history of CV risk, and positive family history of myocardial infarction at the age <50 years.

The following reasons for switching or combining antihypertensive medication or introducing new medication were evaluated in the survey: bad tolerance to the current medication, insufficient BP control (office BP ≥140/90 or ≥130/80 mm Hg in patients with target organ damage and in patients with diabetes), nonadherence or noncompliance with current medication, increased cardiovascular risk of the patient, type 2 diabetes mellitus, aiming for a better 24-hour effect, or other reasons. GPs could choose one or more reasons for switching medication for each patient.

### Data Analysis

The main aim of the survey was to assess reasons regarded by the physician as the most important to prompt a switch of antihypertensive medication. Patients’ current antihypertensive treatment, presence of cardiovascular and metabolic risk factors, and the rate of BP control according to the European Society of Hypertension/European Society of Cardiology guidelines,^7^ that is, to determine how many patients had office BP values <140 mm Hg systolic and <90 mm Hg diastolic was evaluated. Furthermore, the severity of hypertension (mild hypertension with systolic BP 140–159 mm Hg and diastolic BP 90–99 mm Hg; moderate hypertension with systolic BP 160–179 mm Hg and diastolic BP 100–109 mm Hg; severe hypertension with systolic BP ≥180 mm Hg and diastolic BP ≥110 mm Hg) was determined.

Collection and analysis of data was performed by an independent agency Cegedim CZ. Because of the descriptive nature of the results, no statistical test was applied to the data collected.

## RESULTS

Two hundred eighty-six physicians—GPs from all regions of the Czech Republic participated in the survey. They surveyed a total of 4341 hypertensive patients. The mean age of the patients was 59.8 years (median 60.0 years). The distribution of age subgroups is shown in Table 1. A total of 51.5% were men and 46.0% women, in 2.5% gender was not identified in the questionnaires.

**TABLE 1:**
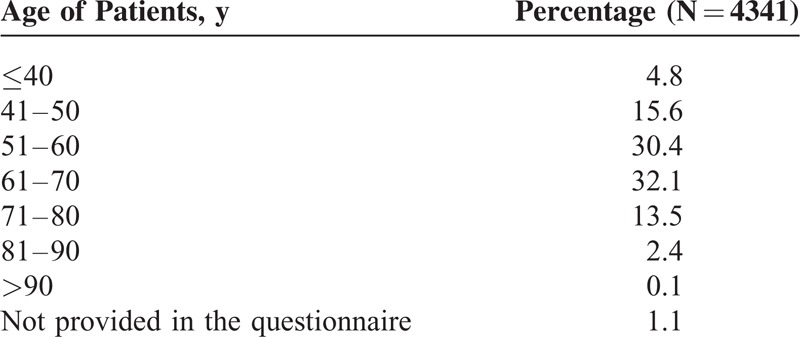
Distribution of Different Age Subgroups in the Survey

Office systolic BP was controlled (<140 mm Hg) in 10.4% of patients and diastolic BP (<90 mm Hg) in 18.5% of patients, respectively. BP control rate according to office BP values is shown in Table 2. The majority of patients had mild hypertension: 47.8% had systolic BP within the range of 140 to 159 mm Hg and 49.7% diastolic BP within 90 to 99 mm Hg.

**TABLE 2:**
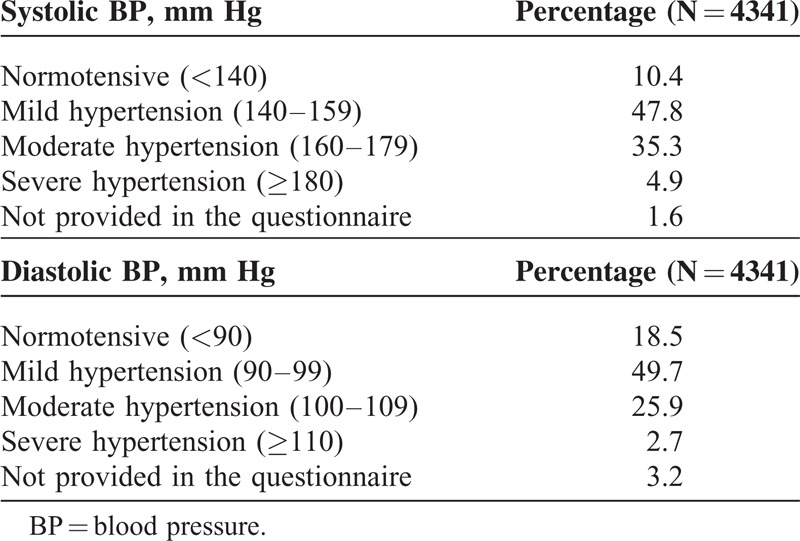
Blood Pressure Control

Only 16.5% of patients had normal weight with BMI <25 kg/m^2^. Overweight (BMI 25–29.9 kg/m^2^) was present in 43.1%, obesity (BMI >30 kg/m^2^) in 25.8% of patients. In 14.6% of patients, data on BMI were not provided by physicians in the questionnaires.

Prevalence of cardiovascular risk factors and target organ damage is shown in Table 3. Overweight or obesity and hypercholesterolemia were present in the majority of patients. About one third of patients had diabetes, smoking habit, or had positive family history of CV events (Table 3). A vast majority of patients (96.3%) had a history of at least 1 cardiovascular risk factor or target organ damage.

**TABLE 3:**
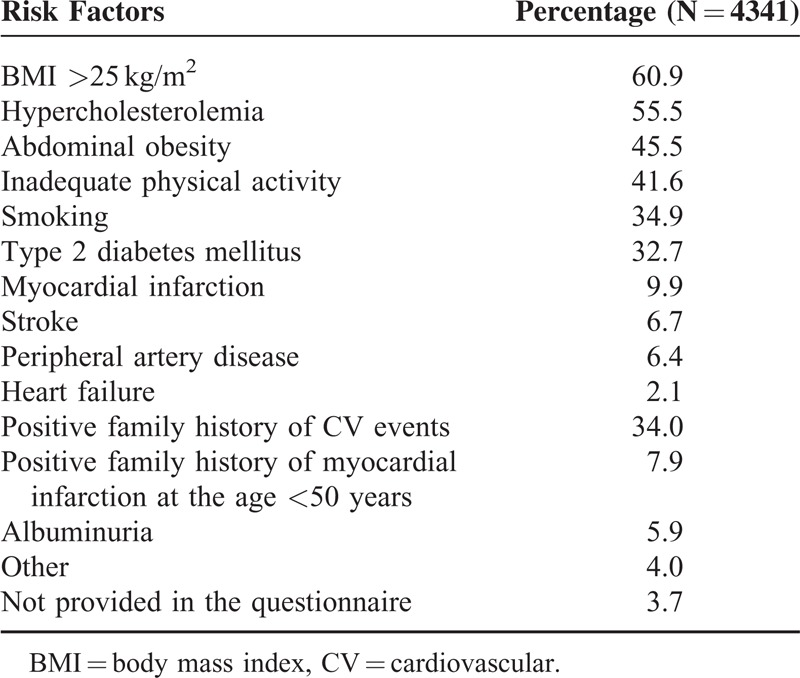
Prevalence of Cardiovascular Risk Factors and Target Organ Damage

**FIGURE 1:**
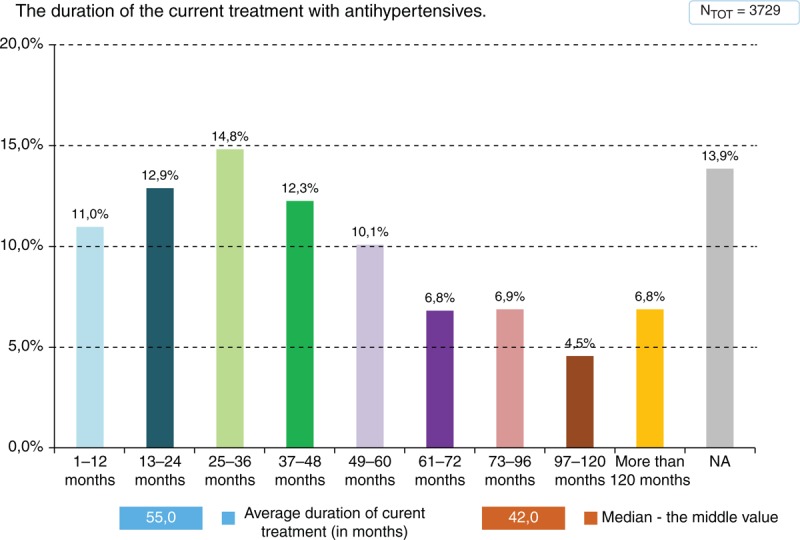
Average duration of current treatment (in months).

A total of 85.9% of patients were treated by antihypertensive drugs for the mean period of 55 months before the survey (median 42 months). The duration of prior antihypertensive treatment is shown in Figure 1. A total of 14.1% of patients did not use any antihypertensive medication at the time of the survey.

Table 4 shows antihypertensive treatment before switching medication. A total 25.9% of patients were on monotherapy with 1 of the 5 major antihypertensive classes according to current guidelines: angiotensin receptor blockers (ARB), angiotensin converting enzyme inhibitors, calcium channel blockers (CCB), beta blockers, and diuretics.^8^ A total of 34.0% of patients used a combination of 2 antihypertensive drugs, 12.6% a combination of 3 drugs, and 2.8% took 4 different antihypertensive drugs (Table 4).

**TABLE 4:**
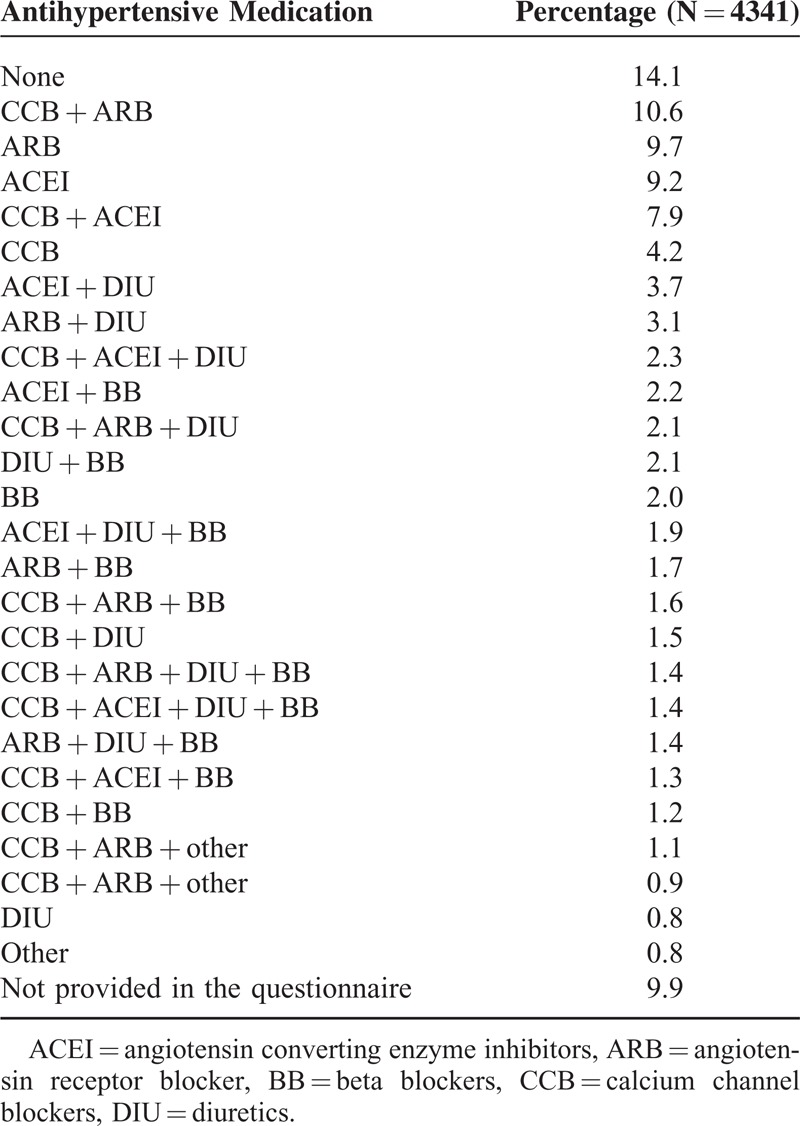
Antihypertensive Medication of the Patients

**FIGURE 2:**
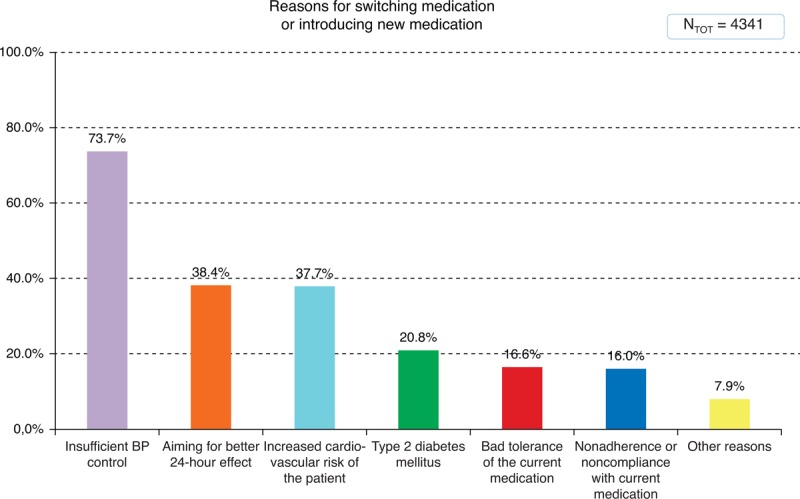
Reasons for switching medication or introducing new medication. BP = blood pressure.

The reasons for switching antihypertensive medication or introducing new medication are shown in Figure 2. The most common reason was insufficient BP control (not reaching BP targets), followed by aiming for a better 24-hour effect and increased cardiovascular risk of the patient.

## DISCUSSION

This large nationwide survey shows that in current general practice the most common reason for switching antihypertensive medication is insufficient BP control.

A survey performed in Italy in the late 90s also identified insufficient BP control as the most frequent cause of treatment switching by doctors (51.2%), followed by adverse drug effects (34.5%).^4^ However, the order of prominence was reversed in the patients’ opinions, with 53% of treatment switches attributed to adverse effects and 34% to inadequate BP control.^4^ Inhibitors of the renin-angiotensin and CCBs were the most often prescribed antihypertensive classes in both our and the Italian survey. The widespread use of ARB in our registry, which were not available a decade ago and are known for their low occurrence of adverse effects, may account for a relatively low need to switch medication because of bad tolerance of the medication (16.6%).

Our survey is also in agreement with results of a recent US study, which showed that failure to achieve BP goals was the most common reason for a change in therapy (68%–74%).^6^ In this study, intolerance of ARBs leading to switching medication was lower compared with that of angiotensin converting enzyme inhibitors (9.5% vs 29.5%, *P* = 0.001) and CCB (21.6%, *P* = 0.03).^6^

Surprisingly, the second most frequently reported reason for medication switch in our survey was insufficient 24-hour effect of the current medication. It is well known that the early morning BP surge is associated with an increase in the incidence of CV events, including stroke and myocardial infarction.^8^ Use of long-acting drugs improves 24-hour BP control and may result in improved clinical outcomes.^8^ When evaluated by ambulatory BP monitoring, the systolic and diastolic BP remains relatively stable with long-acting drugs, even when a patient forgets to take a single or even 2 daily doses.^9^

In patients with arterial hypertension, other cardiovascular (CV) and metabolic risk factors, such as overweight or obesity, hypercholesterolemia, or diabetes mellitus are often present.^3,10,11^ This was also shown in our survey: more than two thirds of patients (68.9%) were overweight or obese and a substantial proportion of patients had hypercholesterolemia or type 2 diabetes. This clustering of multiple comorbidities increases the overall cardiovascular risk of the patient, and should prompt the physician to employ more aggressive hypertension control and risk factor modification.^7^ Unfortunately, in these patients, BP control is more difficult to achieve by antihypertensive treatment^7,12^ and frequent changes of antihypertensive medication are often necessary.

Based on the results of recent large controlled trials, such as Anglo-Scandinavian Cardiac Outcomes Trial or Avoiding Cardiovascular Events through Combination Therapy in Patients Living with Systolic Hypertension Trial,^13,14^ a combination of a calcium channel blocker with a drug interfering with the renin-angiotensin system is usually preferred in patients with diabetes mellitus or in those with an accumulation of other metabolic risk factors, which was also observed in our survey.

Various patient characteristics have been associated with uncontrolled hypertension, including older age, obesity, lack of exercise, race/ethnicity, access to health care, and poor compliance.^15^ Other important factors are older age, multidrug regimens, lack of knowledge by the patient of their target systolic BP and antihypertensive adverse drug effects.^16^ Patient characteristics in our survey confirm that such patients are more likely to have difficult-to-control hypertension.

Treatment adherence can be substantially improved by a simplification of the therapeutic regime, that is, by reducing the number of tablets used. For this purpose, fixed combinations of antihypertensive drugs are a very helpful tool offering greater and faster antihypertensive effect and lesser occurrence of adverse effects.^17,18^ A meta-analysis of 9 clinical trials comparing fixed combinations to free combinations of the same drugs found that the use of a fixed combination improved patient adherence by 26%.^19^

In conclusion, we found that the major reason for switching antihypertensive treatment in general practice is insufficient BP control. Further research is warranted to explore whether new treatment modalities aimed to improve BP control, such as the use of fixed combinations, bring the desired reduction of cardiovascular morbidity and mortality.
